# Psychosocial Factors Linked to Uncontrolled Infection and Mortality among People Living with HIV Who Use Substances: A Latent Class Analysis

**DOI:** 10.1007/s10461-024-04410-2

**Published:** 2024-08-02

**Authors:** Renae D. Schmidt, Viviana E. Horigian, Rui Duan, Sharleen T. Traynor, Carly A. Davis, Sophia T. Gonzalez, Derrick J. Forney, Raul Mandler, Carlos Del Rio, Lisa R. Metsch, Daniel J. Feaster

**Affiliations:** 1 Department of Public Health Sciences, University of Miami Miller School of Medicine, 1120 Northwest 14th Street, Miami, FL 33136 USA; 2 Clinical Trials Research Associate Program, Durham Technical Community College, Durham, NC 27703 USA; 3 Division of Therapeutics and Medical Consequences, National Institute on Drug Abuse, National Institutes of Health, Bethesda, MD 20892 USA; 4 Division of Infectious Diseases, Department of Internal Medicine, Emory University, Atlanta, GA 30322 USA; 5 Department of Sociomedical Sciences, Mailman School of Public Health, Columbia University, 722 West 168th Street, New York, NY 10032 USA

**Keywords:** HIV, Substance use disorder, Mental health, Co-occurring conditions

## Abstract

To determine whether endorsement patterns of psychosocial symptoms revealed distinct subgroups, or latent classes, of people living with HIV who use substances (PLWH-SU), and to assess whether these classes demonstrated differential health outcomes over time. This study uses data from 801 PLWH-SU initially enrolled across 11 US hospitals during 2012–2014 and followed up in 2017. Latent class analysis included 28 psychosocial items. Regression analysis examined class membership as a predictor of viral suppression. Survival analysis examined class as a predictor of all-cause mortality. The selected model identified five unique classes. Individuals in classes characterized by more severe and more numerous psychosocial symptoms at baseline had lower likelihoods of viral suppression and survival. The study demonstrated the importance of considering patterns of overlapping psychosocial symptoms to identify subgroups of PLWH-SU and reveal their risks for adverse outcomes. Integration of primary, mental health, and substance use care is essential to address the needs of this population.

## Introduction

Human immunodeficiency virus (HIV) remains a leading public health issue in the U.S. with an estimated 1.2 million people living with HIV (PLWH) [1]. While there have been remarkable advances in HIV treatment, PLWH carry a disproportionate burden of comorbid mental health disorders including psychotic disorders, bipolar disorders, recurrent major depressive disorder, and mood disorders, which can complicate HIV management and access to treatment [2, 3]. Substantial evidence has linked mental health impairment with negative health outcomes along the HIV care continuum including elevated viral load, decreased CD4+ levels, increased opportunistic illnesses, and mortality [3]. Therefore, meeting needs of PLWH entails identifying and addressing mental health problems among this population.

Of particular concern among PLWH is substance use disorder (SUD). Nearly half of PLWH report a SUD [4]. Substance use is both a significant driver of the HIV epidemic and is associated with poor outcomes among PLWH [4]. Like other mental health conditions, SUD can have deleterious impacts on HIV progression as PLWH who use substances (PLWH-SU) are less likely to access care, adhere to antiretroviral treatment plans, and reach viral suppression compared to PLWH that do not use substances [5]. Further, SUD and additional psychiatric conditions often co-occur, resulting in severe illness, disability, and poor treatment outcomes among people who use substances [6]. Therefore, PLWH-SU burdened with comorbid mental health issues are especially vulnerable to mismanaging their intersecting conditions.

Evidence has linked psychosocial factors among individuals with HIV and with SUD to numerous adverse behaviors and outcomes. For example, depression and generalized anxiety disorder have been associated with decreased HIV medical adherence, poor viral load outcomes, increased hospitalizations, and mortality among PLWH [7, 8]. People who use substances who have anxiety and depression also experience more severe drug use [6]. PLWH and individuals who use substances face higher risk of suicidal ideation, attempts, and death compared to the general population, and suicidal thoughts have been linked to negative HIV-related outcomes, including antiretroviral treatment discontinuation, engagement in risky behavior, and unsuppressed viral load [9–11]. Past trauma, including abuse, violence, and discrimination, can interfere with HIV diagnosis and treatment [12]. Discrimination is also associated with more severe substance use and lack of engagement in substance use treatment [13, 14]. Meanwhile social support has been linked to retention in treatment among people with SUD, and moderates the relationship between SUD with medication adherence and viral load among black women with HIV [15, 16].

The complex intersectionality of HIV, substance use, and psychosocial symptomatology poses a threat to health and wellbeing. Therefore, it is critical to identify psychosocial factors among PLWH-SU which could affect health outcomes. One approach is to differentiate profiles of individuals based on patterns of reported psychosocial symptoms. This can be done via a latent class analysis (LCA), a person-centered mixture modeling technique which identifies latent subpopulations based on patterns of responses to observed variables, assuming that membership in these unobserved subpopulations—or classes—can explain patterns of assessment indicators [17]. LCA offers the opportunity to unveil distinct patterns of multiple characteristics which may provide a more detailed understanding of the phenomena which occur among different individuals. Researchers have used LCA to better understand behaviors and outcomes among populations living with or at risk of exposure to HIV, including defining latent classes of stress to better understand risk and protective factors for HIV among gay and bisexual men [18] and among justice-involved heterosexual couples [19], characterizing patterns of social determinants of health to predict substance use among women living with HIV [20], exploring patterns of polysubstance use among PLWH to assess the relationship with financial hardship, incarceration, homelessness, and mental health [4], and classifying PLWH-SU based on latent profiles of barriers to care to assess differential intervention effects [21].

In this study, LCA was applied to classify PLWH-SU based on item-level psychosocial symptoms. Regression and survival analyses were then applied to assess whether subgroups experienced differential viral suppression and mortality. Instead of assessing individual symptoms or behaviors, like suicidality alone, this analysis considers nuanced combinations of several factors which may have a compounded impact on these individuals and influence the likelihood to experience critical outcomes with the objective of illuminating commonalities and differences across individuals that have implications for practice and future research.

## Methods

### Study Sample

This is a secondary data analysis of participants enrolled across two randomized controlled trials conducted by the National Institute on Drug Abuse National Drug Abuse Treatment Clinical Trials Network (CTN). During 2012–2014, 801 inpatient PLWH-SU across 11 U.S. hospitals participated in CTN0049, which tested the efficacy of patient navigation and financial incentives for achieving viral suppression among a patient population meeting at least 1 of 3 HIV-related criteria (i.e. had an AIDS-defining illness, had a CD4 cell count < 350 cells/µL and a viral load > 200 copies/mL within the past 6 months, or had a CD4 count ≤ 500 cells/µL and a viral load > 200 copies/mL within the past 12 months) [22]. Participants were randomly assigned to receive patient navigation alone, patient navigation plus financial incentives, or treatment as usual. Participant eligibility included being 18 + years old and HIV-positive, with reported or medical record documentation of any opioid, stimulant, or heavy alcohol use in the past 12 months. Participant data during CTN0049 were captured at baseline, 6 months, and 12 months.

Then, in 2017, 422 of the individuals enrolled in CTN0049, were rescreened for a second trial, CTN0064, which tested the efficacy of an intervention for PLWH-SU with HCV/HIV coinfection and also assessed the long term follow up of CTN0049 [23]. The final sample size of the current study includes all 801 participants randomized into CTN0049, of which 422 had data at baseline CTN0064, 243 died and the other 136 participants were lost to follow up.

### Classification Items

Participant responses to psychosocial items capturing baseline depression, anxiety, suicidal ideation, despair, social support, and past trauma were included in the LCA. Each response was converted to a binary format, with 0 indicating the absence and 1 indicating the presence of a negative psychosocial factor.

The Brief Symptom Index-18 (BSI-18), was designed to assess dimensions of psychological distress [24]. Twelve of 18 items were included in the analysis: participant responses to six items assessing depression and six items assessing anxiety on a five-point scale according to how much they had been bothered by symptoms the prior week. Responses *Not at all* and *A little bit* were coded as 0; *Moderately*, *Quite a bit*, and *Extremely* were coded as 1.

The Concise Health Risk Tracking Self-Report (CHRT-SR) is a 12-item self-report instrument used to assess suicidal ideation and propensity [25]. Participants responded to three items assessing despair, three items assessing suicidal ideation, and two items assessing lack of social support on a four-point scale according to how much they had been bothered by feelings the prior week. Responses *Strongly disagree, Disagree,* and *Neutral* were coded as 0 and *Strongly agree* and *Agree* were coded as 1.

Five Short Social Support Scale (SSS) items assessed tangible social support for PLWH [26]. Respondents are asked, "How often were each of the following kinds of support available to you if you needed it in the last four weeks?", and can respond on a five-point scale. To capture the lack of support, responses *All of the time, Most of the time,* and *Some of the time* were coded as 0 and *None of the time* and *A little of the time* were coded as 1.

Past trauma encompassed a history of abuse, violence, and/or discrimination. Participants indicating experiences of physical attacks, beatings, sexual abuse, or rape, either as a child or an adult, were included in the analysis as having an abuse history (coded as 1). Interpersonal violence (IPV) was evaluated by three "yes/no" items from an adapted IPV screening tool [27]. Participants affirming that they had been either threatened or controlled by a sexual partner, or that their sexual partner ever threw, broke, or punched things, were considered to have an IPV history (coded as 1). Additionally, history of discrimination in healthcare settings was assessed by the Medical Mistrust scale [28]. Participants were asked if they had ever encountered discrimination or feelings of inferiority due to their HIV status, gender, sexual orientation, race/ethnicity, or drug use. Affirmative responses to any of these reasons were treated as a history of discrimination (coded as 1).

### Outcomes

Two critical health outcomes were assessed for each identified class. Viral suppression was defined as having an HIV viral load ≤ 200 copies/mL versus > 200 copies/mL and was assessed at baseline, 6-months, and 12-months during CTN0049 and at long term follow up, approximately five years later. Mortality was determined by reviewing the primary cause of death in the trial death form or extracted from the National Death Index.

### Covariates

Several variables, captured at baseline of CTN0049, were enumerated by class and included as covariates in analyses. These included self-reported age, gender, race, and ethnicity and recent incarceration. Further, due to established impact of social determinants of health on outcomes of HIV and SUD, geographic residence, housing instability, food insecurity, and recent incarceration were also included. Because the South experiences the highest burden of HIV incidence and mortality and trails behind in providing quality care, southern/ non-southern geographic residence was included as a binary variable determined by the study site location [29]. Participant living situations over the past six months were assessed for housing instability. Participants that reported being homeless or living in a shelter, permanent single-room occupancy hotel, HIV/AIDS group room, transitional housing, or other residential facility or institutions were classified as having unstable housing status. Food insecurity was assessed using the Household Food Insecurity Access Scale, a 9-item questionnaire assessing various food insecurity domains in the past 4 weeks. Total scores ranged from 0 to 27, with higher scores representing greater food insecurity [30]. Finally, psychiatric history was based on diagnoses of mental health conditions during initial CTN0049 hospitalization or seeing a professional to address mental health problems in the past 6 months.

As substance use is associated with negative outcomes among PLWH, three variables on baseline substance use behaviors were assessed among participants as well [4–6]. These included (1) days endorsing substance use as determined by the drug and alcohol use module of the Addiction Severity Index-Lite—a structured clinical interview which captures substance use over the past 30 days, (2) the Alcohol Use Disorders Identification Test (AUDIT) assessing alcohol consumption, drinking behaviors, and alcohol-related problems, and (3) the Drug Abuse Screening Test (DAST) assessing drug use and drug use problems, not including alcohol or tobacco use, in the past 12 months [31–33].

### Analytic Plan

After each of the 28 classification items was operationalized, LCA was conducted to identify classes based on patterns of reported symptomatology. Models with two to six classes were estimated using robust maximum likelihood and were evaluated based on several fit indices including the Akaike Information Criterion (AIC), the Bayesian Information Criterion (BIC), the sample size adjusted Bayesian Information Criterion (ssaBIC), entropy, and the Lo–Mendell–Rubin Likelihood Ratio Test (LMR‐LRT) [34, 35]. Additionally, estimated probabilities, plot/plot interpretability, and sample size of each class were considered in selection of the final model. A confirmatory factor analysis (CFA) was performed to verify responses from specific items were consistent with their intended measurements (BSI, CHRT, and SSS).

Descriptive statistics were calculated for each group of individuals identified by most likely latent class membership. ANOVA and Chi-Square tests were performed to assess differences between classes. A generalized linear mixed model (GLMM) was used to examine the impact of class membership on viral suppression over time. An initial model testing the interaction between visit and class membership revealed no significant interaction, and the interaction term was removed. Visit was included as a covariate to account for changes over time, and a random intercept was included for the participants nested within the site, to account for similarity across individuals within each site. Survival analysis using accelerated failure time model with log-logistic distribution examined class membership as a predictor of survival over time. A random term was included within both models to account for correlated data by site. While LCA addresses missing data via maximum likelihood estimates, missing data were excluded from final analyses as the GLMM ignores any observation with a missing value for any variable. The LCA and CFA were conducted using Mplus 6.1 [36]. All other analyses were conducted using SAS University Edition [37]. For all analyses, two-tailed p-values less than 0.05 were considered statistically significant.

## Results

### Characteristics of the Study Population

Among the overall sample of 801 participants, 67.4% were male, 72.0% were Black/African American individuals, 12.0% were White individuals, and 11.0% were Hispanic individuals. The average age was 44.2 years (SD 10.0). There were slightly more participants enrolled in southern sites (59.2%). Approximately 22.0% had a recorded psychiatric history, 37.2% were unstably housed, and 6.4% were recently incarcerated. The average food insecurity score was 6.21 (SD = 7.9). A detailed description of participant characteristics can be found in the study’s primary outcomes publication [22].

### Psychosocial Latent Classes

LCA model fit was assessed for models with 2–6 classes (Table 1). Multiple fit statistics and interpretability indicated that a 5‐class model (bolded) best fit the data. Both the BIC score and the sample‐size Adjusted BIC score were lower in the 5‐class model than the 2-, 3-, or 4-class models, while maintaining a high entropy. The 5‐class model also presented a solution with a logical substantive interpretation, adequate class distinction and sample sizes; whereas the 6-class model had classes with fewer than 10%.

**Table 1:** Latent class analysis model fit statistics

Model	Log likelihood	AIC	BIC	ssaBIC	LMR-LRT	LMR-LRT p-value	Entropy
2-class	− 10,812.465	21,738.929	22,006.023	21,825.016	3244.608	< 0.0001	0.916
3-class	− 10,421.468	21,014.936	21,417.92	21,144.822	777.98	0.0004	0.889
4-class	− 10,163.106	20,556.212	21,095.086	20,729.897	514.073	0.0034	0.88
**5-class**	− **10,015.519**	**20,319.037**	**20,993.801**	**20,536.521**	**293.66**	**0.0704**	**0.876**
6-class	− 9890.184	20,126.368	20,937.022	20,387.651	249.383	0.0785	0.892

AIC: Akaike Information Criterion; BIC: Bayesian Information Criterion; LMR-LRT: Lo–Mendell–Rubin likelihood ratio test; ssaBIC: sample size adjusted Bayesian Information Criterion

**Fig. 1:**
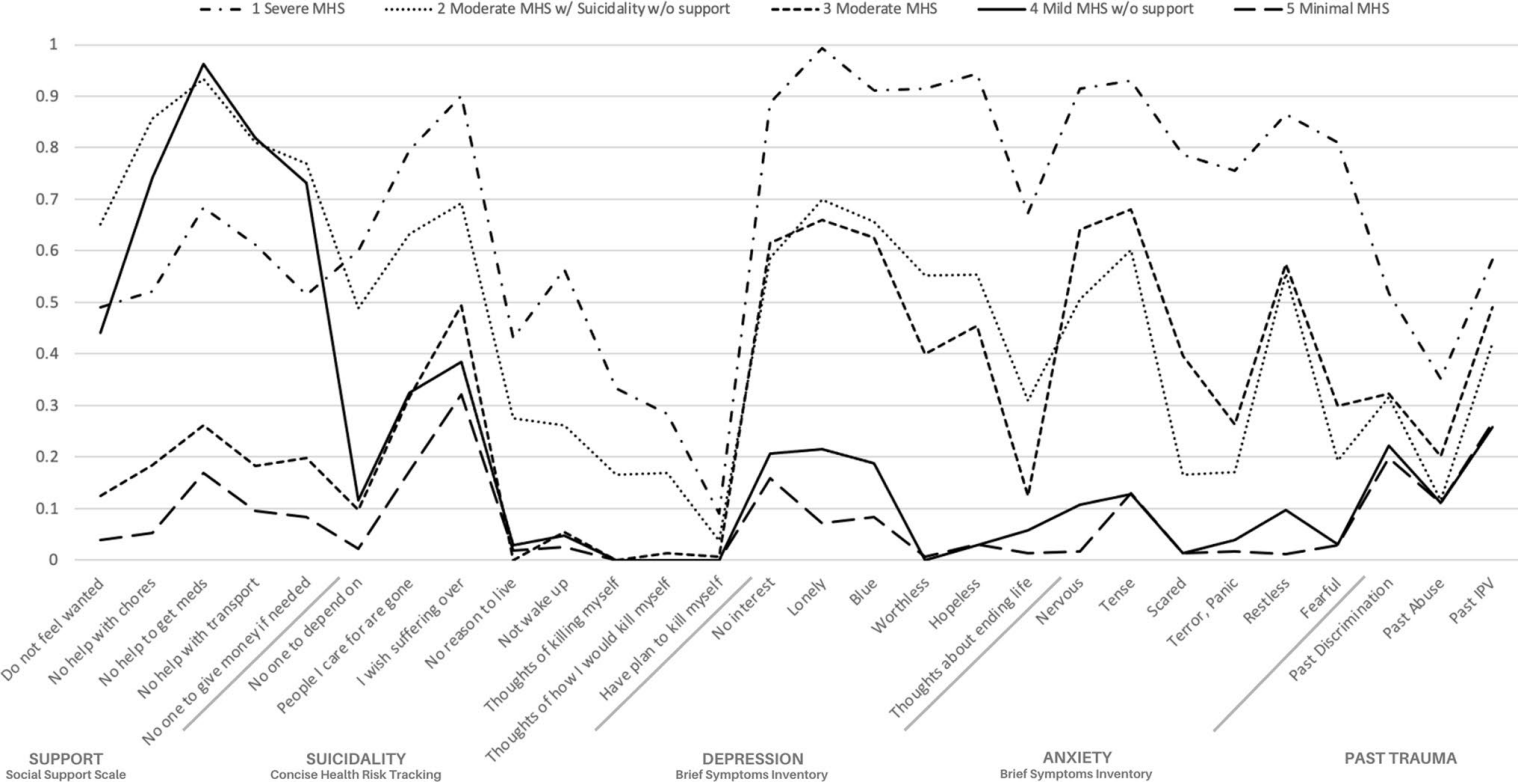
Probabilities of class membership for a five‐class solution of item-level psychosocial factors. *MHS: Mental Health Symptomatology

The selected model presents five “classes” of individuals with unique baseline psychosocial symptomatology. These classes were labeled (1) *Severe mental health symptomatology (MHS),* (2) *Moderate MHS w/suicidality and w/o support,* (3) *Moderate MHS,* (4) *Mild MHS w/o support,* and (5) *Minimal MHS.* Figure 1 shows the probabilities of endorsing each item by most‐likely class membership.

Class 1—the *Severe MHS* class—comprised 12.9% of the sample (N = 103), the lowest proportion of participants across classes. This class was characterized by the highest probabilities of endorsing each psychosocial item within the constructs of depression, anxiety, suicidal thoughts, despair, and past trauma, as compared to the other classes. The only exception to this pattern was in the five social support items, which were the third highest in this class following the two classes named specifically for their lack of support. There were particularly high probabilities of endorsing depression items, anxiety items, and items indicating despair. Substance use behaviors (reporting an average of 16.7 substance use days out of the past 30 via the ASI at baseline) and severity (mean AUDIT score 12.9; mean DAST score 6.4) were highest among the Severe MHS class. Class 2—the *Moderate MHS w/suicidality and w/o support* class—comprised 15.6% of the sample (N = 128). This class was characterized by the highest probabilities of endorsing a lack of social support and the second highest probabilities of endorsing items of suicidal thoughts, despair, and depression. The second highest average substance use days (12.5 out of 30) and AUDIT score (9.7) were found among member of this class as well. Class 3—the *Moderate MHS* class—comprised 21.6% of the sample (N = 173). This class was characterized by the second highest probabilities of endorsing items of anxiety, indicating they feel “nervous”, “tense”, “scared”, “panic”, “restless”, and “fearful”. They also had the second highest probability of endorsing each past trauma. Notably, they had the second lowest probability to endorse lacking social support. Class 4—the *Mild MHS w/o support* class—comprised 20.5% of the sample (N = 164). This class was characterized by the lowest or second lowest probabilities of endorsing each psychosocial item except for social support, which paralleled Class 2’s high probabilities of endorsing lack of support, particularly help with getting medication. This class had negligible probability of endorsing suicidal thoughts, and probabilities less than 10% of endorsing items of anxiety or despair. Class 5—the *Minimal MHS* class—comprised 29.1% of the sample (N = 233), the highest proportion of participants across classes. This class was characterized by the lowest probabilities of endorsing each psychosocial item, particularly lacking social support. The Minimal MHS class also had the lowest average days reporting substance use (9.9 days of out of 30) and lowest mean DAST score (3.6). Participant characteristics by class are displayed in Table 2.

**Table 2:** Participant characteristics by Class

	Class 1 severe MHS	Class 2 moderate MHS w/suicidality and w/o support	Class 3 moderate MHS	Class 4 mild MHS w/o support	Class 5 minimal MHS	Test of significant difference	p-value
Total	103 (12.9%)	128 (16.0%)	173 (21.6%)	164 (20.5%)	233 (29.1%)		
Age	44.5 (9.2)	44.4 (10.3)	42.9 (10.2)	47.4 (9.6)	44.2 (10.0)	F = 4.63	< 0.01
Gender							
Female	39 (37.9%)	34 (26.6%)	71 (41.0%)	49 (29.9%)	68 (29.2%)	*X*^2^ = 10.82	0.03
Male	64 (62.1%)	94 (73.4%)	102 (59.0%)	115 (70.1%)	165 (70.8%)		
Race/Ethnicity							
Black/Afr Am	62 (60.2%)	90 (70.3%)	125 (72.3%)	130 (79.3%)	170 (73.0%)	*X*^2^ = 19.68	0.07
Hispanic	19 (18.4%)	13 (10.2%)	16 (9.2%)	15 (9.1%)	25 (10.7%)		
Other	5 (4.9%)	6 (4.7%)	13 (7.5%)	8 (4.9%)	8 (3.4%)		
White	17 (16.5%)	19 (14.8%)	19 (11.0%)	11 (6.7%)	30 (12.9%)		
Treatment arm assignment							
PN	38 (36.9%)	45 (35.2%)	49 (28.3%)	52 (31.7%)	82 (35.2%)	*X*^2^ = 5.65	0.69
PN + CM	33 (32.0%)	44 (34.4%)	63 (36.4%)	50 (30.5%)	81 (34.8%)		
TAU	32 (31.1%)	39 (30.5%)	61 (35.3%)	62 (37.8%)	70 (30.0%)		
Psychiatric history							
Yes	44 (42.7%)	39 (30.5%)	45 (26.0%)	34 (20.7%)	39 (16.7%)	*X*^2^ = 29.38	< 0.0001
Geographic residence							
North	54 (52.4%)	55 (43.0%)	65 (37.6%)	70 (42.7%)	83 (35.6%)	*X*^2^ = 9.59	0.05
South	49 (47.6%)	73 (57.0%)	108 (62.4%)	94 (57.3%)	150 (64.4%)		
Housing instability							
Yes	43 (41.7%)	72 (56.3%)	64 (37.0%)	67 (40.9%)	52 (22.3%)	*X*^2^ = 43.82	< 0.0001
Recent incarceration							
Yes	9 (8.7%)	9 (7.0%)	7 (4.0%)	11 (6.7%)	15 (6.4%)	*X*^2^ = 2.63	0.62
Food insecurity	12 (9.4)	9.1 (8.6)	6.7 (7.7)	4.4 (6.1)	3.0 (5.8)	F = 35.31	< 0.0001
AUDIT	12.9 (12.3)	9.7 (9.6)	8.7 (10.1)	7.8 (8.1)	8.1 (8.1)	F = 5.78	< 0.0001
DAST	6.4 (2.7)	5.0 (2.8)	5.4 (2.7)	4.1 (2.9)	3.6 (2.8)	F = 23.61	< 0.0001
Substance use days	16.7 (11.9)	12.5 (11.2)	11.6 (11.5)	11.5 (11.8)	9.9 (11.0)	F = 6.50	< 0.0001
Viral suppression							
Baseline	15 (14.6%)	13 (10.2%)	19 (11.0%)	13 (7.9%)	27 (11.6%)	*X*^2^ = 3.11	0.54
6 months	44 (42.7%)	53 (41.4%)	63 (36.4%)	50 (30.5%)	108 (46.4%)	*X*^2^ = 12.61	0.01
12 months	39 (37.9%)	44 (34.4%)	54 (31.2%)	63 (38.4%)	92 (39.5%)	*X*^2^ = 8.58	0.07
Follow up	18 (17.5%)	21 (16.4%)	20 (11.6%)	33 (20.1%)	46 (19.7%)	*X*^2^ = 7.92	0.09
Death	39 (37.9%)	39 (30.5%)	47 (27.2%)	50 (30.5%)	68 (29.2%)	*X*^2^ = 3.73	0.44

N and % shown for categorical variables, mean and standard deviations shown for continuous variables

Tests for significant differences by class include One-way ANOVA (denoted by F) and Chi-Square Test (denoted by *X*^2^)

As shown in Table 2, there was a significant difference in proportion of virally suppressed individuals across the five classes at only one time point: 6 months (*X*^2^ = 12.61; df = 4; p = 0.01). The highest proportion of virally suppressed individuals at baseline was in the *Severe MHS* class (14.6%), while the *Minimal MHS* class had the highest proportions at 6 months (46.4%) and 12 months (39.5%). The highest proportion at long term follow up was found in the *Mild MHS w/o support* class (20.1%), though they had the lowest proportion at baseline (7.9%) and 6 months (30.5%). Results of the GLMM analysis (Table 3) demonstrated that class membership was associated with viral suppression over time. Those in the *Moderate MHS* class were less likely to be virally suppressed—on average, across multiple follow ups—as compared to those in the *Minimal MHS* class (OR = 0.62; 95% CI 0.44–0.88; p = 0.01). Black/African American individuals as compared to White individuals (OR = 0.55; 95% CI 0.38–0.80; p ≤ 0.01) and individuals living in southern states as compared to non-southern states (OR = 0.59; 95% CI 0.46–0.75; p ≤ 0.0001) were less likely to be virally suppressed. Individuals with a psychiatric history were more likely to be virally suppressed (OR = 1.44; 95% CI 1.09–1.90; p = 0.01).

**Table 3:** Results of 1) generalized linear mixed model analysis examining class membership as a predictor of viral suppression and 2) parametric survival analysis examining class membership as a predictor of survival

	Reference group	Viral suppression		Survival	
		Odds ratio (95% CI)	p-value	Time ratio (95% CI)	p-value
Age		1.02 (1.01–1.03)	0.00	0.97 (0.95–0.99)	0.01
Class					
Severe MHS	Minimal MHS	0.81 (0.53–1.26)	0.35	0.62 (0.39–0.99) *[Severe MHS* *vs all others]*	0.04
Moderate MHS w/suicidality & w/o support		0.73 (0.49–1.07)	0.11		
Moderate MHS		0.62 (0.44–0.88)	0.01		
Mild MHS w/o support		0.72 (0.51–1.02)	0.07		
Female	Male	1.05 (0.81–1.36)	0.71	1.19 (0.85–1.67)	0.30
Race/Ethnicity					
Black/Afr Am	White	0.55 (0.38–0.80)	0.00	1.07 (0.66–1.72)	0.78
Hispanic		0.67 (0.40–1.10)	0.11	1.51 (0.77–2.98)	0.23
Other		0.73 (0.39–1.39)	0.34	1.85 (0.77–4.44)	0.17
Treatment Arm					
PN	TAU	1.13 (0.84–1.52)	0.42	0.91 (0.63–1.32)	0.61
PN + CM		1.22 (0.91–1.64)	0.18	1.17 (0.80–1.72)	0.41
Food insecurity		1.00 (0.98–1.01)	0.63	1.00 (0.97–1.02)	0.78
Housing instability	No	0.82 (0.63–1.08)	0.16	0.83 (0.59–1.16)	0.26
Recent incarceration	No	1.06 (0.65–1.74)	0.80	2.12 (0.96–4.68)	0.06
Southern residence	No	0.59 (0.46–0.75)	< 0.0001	0.61 (0.40–0.93)	0.02
Psychiatric history	No	1.44 (1.09–1.90)	0.01	1.96 (1.32–2.91)	0.00

There were 243 total deaths across the five classes and no significant difference in proportion of deaths by class (Table 2). The highest proportion of deaths was found in the *Severe MHS* class (37.9%), followed by the *Moderate MHS w/ suicidality and w/o support* and *Mild MHS w/o support* classes (30.5% died in both classes). The lowest proportion of deaths was in the *Moderate MHS* class (27.2%). Survival analysis including each of the five classes revealed no significant differences between class and likelihood of survival. However, when comparing the *Severe MHS* class to the other four classes (Table 3), those in the *Severe MHS* class were less likely to survive [Time Ratio (TR) = 0.62; 95% CI 0.39–0.99; p = 0.04]. Older individuals were also less likely to survive (TR = 0.97; 95% CI 0.95–0.99; p = 0.01), while those with a psychiatric history were more likely to survive (TR = 1.96; 95% CI 1.32–2.91; p < 0.01).

## Discussion

This work aimed to expand our understanding of the critical intersection of HIV, substance use, and mental health by using LCA to identify patterns of psychosocial symptoms among a sample of PLWH-SU recruited during an HIV-related hospitalization. Consistent with existing literature, we found individuals in classes characterized by more serious and more numerous psychosocial symptoms at baseline were at risk for experiencing worse outcomes over time [7, 38]. Individuals in the *Severe MHS* class, demonstrating high probabilities of endorsing a dangerous combination of suicidal, depressive, and anxious symptoms and reported trauma were less likely to survive than all other classes combined. Also, those in the *Moderate MHS* class were less likely to be virally suppressed as compared to the *Minimal MHS* class, indicating that even moderate mental health symptomatology can deter the effectiveness of intervention efforts aimed at achieving viral suppression and should be addressed to ensure healthy patient outcomes. These findings align with previous research indicating that PLWH with mental health symptoms are at an increased risk of having a detectable viral load and a higher mortality rate [7, 38].

While we expected to also observe this relationship in viral suppression among the *Severe MHS* and *Moderate MHS w/ suicidality & w/o support* classes, the significant relationship found only in the *Moderate MHS* class may have emerged due to its larger sample size. This analysis was not designed to detect differences between all classes, rather significant relationships with viral suppression as compared the reference group of the *Minimal MHS* class only. However, it is notable that the ORs for the other three classes fall within the CI of the *Moderate MHS* class in this model. It is important to draw attention here to the low rate of viral suppression in the *Minimal MHS* reference group, which makes it more challenging to uncover significant differences between that and other classes. The low rates of viral suppression among all groups, including the *Minimal MHS* class, may likely be a consequence of larger sociostructural factors at play. As found in the primary outcomes of the CTN0049 trial, only slightly more than a third of participants achieved viral suppression at 12 months, regardless of the intervention [22]. The study’s Lead Investigators mentioned a lack of substance use disorder treatment options could have impacted results, and that perhaps the intensive, individual-level intervention was insufficient to improve HIV outcomes among populations facing systemic and structural barriers.

This study revealed other noteworthy relationships. As supported by national data, Black/African American individuals were less likely to be virally suppressed as compared to White individuals, and individuals living in southern states were less likely to be virally suppressed as compared to non-southern states [29, 39]. Surprisingly, individuals with psychiatric history were more likely to be virally suppressed and to survive. This may be attributed to variable operationalization of “psychiatric history”; 87% of those categorized with psychiatric history were identified by engagement in psychiatric care, versus 13% who had reported comorbid psychiatric conditions at intake. Engagement in psychiatric care may be s a protective factor against poor HIV outcomes and highlights the positive effects of screening for and treating mental health conditions among this population. The promise of engagement in care to mitigate negative HIV outcomes is supported in findings of Traynor and colleagues, who conducted a LCA among the same sample of PLWH-SU and found that those in profiles with lower barriers to care showed a greater response to the patient navigation intervention and had higher rates of 6- and 12-months engagement in care and viral suppression than the treatment as usual group [21]. Among those barriers to care were alcohol and substance use severity measured by the AUDIT and DAST. We similarly found higher substance use severity—as well as higher average days endorsing substance use—among the more severe psychosocial classes with worse outcomes. The impact of substance use on psychosocial factors may reasonably be compounded in these subgroups, and playing a role in some of the associations we see. Further research looking into the relationship with substance use behaviors, class membership, and outcomes over time is warranted.

There are important strengths to this work. While previous studies have demonstrated the utility of LCA to characterize subgroups and understand risks among PLWH-SU, to our knowledge this is the only study to deploy an item-level LCA of several validated instruments, thereby enhancing interpretation of single continuous assessment scores among a sample of PLWH-SU [4, 21]. Interpreting latent patterns across several psychosocial assessments reveals which and how many factors amidst many reported symptoms may be impacting outcomes. This approach revealed notable patterns across individuals’ symptoms, perhaps most notably that social support emerged a key factor characterizing baseline class membership and subsequent outcomes, even when coupled with distinct combinations of other symptoms. Additionally, given opportunities to assess this hard-to-reach population may be potentially limited outside of a research setting, this study plays a valuable role in revealing the relationship between psychosocial factors assessed during hospitalization and critical outcomes experienced in the future. That said, as factors assessed only during hospitalization may also likely change over time, a further look into these specific psychosocial factors as time-varying predictors may be warranted in order to better understand the rate and magnitude of change, how these changes impact health outcomes, and therefore how often patients’ symptomatology should be assessed.

There are also several limitations to this study. First, given the nature of this secondary analysis, no causality can be assumed. Second, several variables were captured via self-report instruments and are prone to varying introspective abilities, response biases, and social desirability. Third, limitations of the LCA include the potential loss of information recoding continuous indicators into categorical variables and reification, or concluding that latent classes identified in the analysis represent actual individuals in the population. Fourth, classification items were not weighted differently from each other and there was partial overlap of some measures’ content. However, patterns can be interpretated while considering these distinct levels of severity or overlap in intent. Fifth, due to a notably high death rate and loss to follow up between trials, long-term data was missing for 47.3% of the baseline sample. Finally, LCA are post-hoc techniques that generate findings for a priori testing. Findings with a different sample may be very different.

Profiles characterized by several factors such as was done here represent typologies that can help researchers and practitioners understand commonalities and differences across individuals that have implications for practice and future research. For example, the *Severe MHS* class exhibited heightened mortality risk due to compounded psychosocial symptoms and severe substance use. As both elevated depressive symptoms and substance use contribute to suicidal ideation, tailored suicide prevention is crucial for individuals meeting characteristics of this class. In the *Moderate MHS* class, although some individual factors may not raise immediate concerns (for example, social support was particularly high among this group as compared to most others, and they reported no suicidality), the presence of moderate syndemic factors such as depression, anxiety, trauma, and despair requires focused attention for achieving viral suppression. Screening for multiple symptoms can identify those needing integrated intervention, especially as some individuals may not receive adequate support despite experiencing mental health issues impacting HIV outcomes. PLWH are more likely to experience at least one episode of major depression than the general population, for example, so care plans which include mental health care may also demonstrate reductions not only in these many intersecting negative mental health symptoms, but overall quality of life [40].

Given that comorbid psychosocial conditions place PLWH-SU at risk for adverse health outcomes and poor quality of life, integration of universal mental health and substance use screening, as well as the provision of mental health and SUD treatment, into HIV primary care, remains essential [3]. PLWH-SU can benefit from a broad range of mental and behavioral health interventions, including evidence-based treatment for SUD, harm reduction services, and comprehensive, patient-centered HIV care that integrates community-based practitioners and medical providers to provide collaborative therapies [41, 42]. Organized, collective efforts between patients, providers, healthcare systems, and the community overall, ensure PLWH-SU can access an array of needed treatment and services [41]. While approximately 50% of PLWH are enrolled in the Ryan White HIV/AIDS Program, and have opportunities to receive a range of medical services—substance use disorder treatment, mental health services, medications, and support services—those most vulnerable (i.e. multiply diagnosed, unstably housed) remain less likely to achieve viral suppression [43]. This is particularly important as the participants in this study represent a severely at-risk population within the HIV epidemic, characterized by being hospitalized, substance users, predominantly from racial/ethnic minority backgrounds, and confronting adverse social determinants of health including food insecurity and unstable housing. In fact, Shokoohi and colleagues found latent profiles based on social determinants of health were linked to substance use among women living with HIV; and emphasizing the significance of addressing interlinked social determinants and drug use through the course of HIV care and treatment [20]. In a LCA of polysubstance use patterns among African American/Black and Latino PLWH from low socioeconomic backgrounds, Cleland and colleagues showed that those in classes with higher polysubstance use and high-risk substance use were more likely to have mental distress and more likely to be experiencing homelessness [4]. It is paramount to uphold models of care which assess and mitigate all intersecting factors—substance use behaviors, mental health symptoms, sociostructural issues—which threaten the wellbeing of PLWH.

Differentiating profiles of psychosocial symptomatology allowed us to identify and understand unique groups of PLWH-SU among this sample. Further, it highlighted the link between psychosocial factors and serious health outcomes among this group. This LCA approach highlights the importance of identifying combinations of overlapping symptoms to establish profiles of PLWH-SU which may guide the development of targeted, evidence-based risk assessment protocols and intervention strategies for those most at risk. Future research should test strategies to address elevated psychosocial symptoms among PLWH-SU as a path to improved health outcomes and examine ways to overcome barriers to integrated, comprehensive approach to addressing HIV, SUD, and mental health issues.

## Data Availability

CTN 0049 trial data are publicly available on NIDA Data Share: https://datashare.nida.nih.gov/ (accessed on September 15 2022). CTN 0064 data has been approved for upload onto NIDA Data Share and will be publicly available soon.

## References

[1] HIV.gov. U.S. statistics: fast facts. https://www.hiv.gov/hiv-basics/overview/data-and-trends/statistics#:~:text=Approximately%201.2%20million%20people%20in,who%20have%20sex%20with%20men. Accessed 11 Jan 2023.

[2] Gallant J, Hsue PY, Shreay S, Meyer N. Comorbidities among US patients With prevalent HIV infection—a trend analysis. J Infect Dis. 2017;216(12):1525–33. 10.1093/infdis/jix518. 29253205 10.1093/infdis/jix518

[3] Remien RH, Stirratt MJ, Nguyen N, Robbins RN, Pala AN, Mellins CA. Mental health and HIV/AIDS: the need for an integrated response. AIDS. 2019;33(9):1411–20. 10.1097/QAD.0000000000002227. 30950883 10.1097/QAD.0000000000002227 PMC6635049

[4] Cleland CM, et al. African American/Black and Latino adults with detectable HIV viral load evidence substantial risk for polysubstance substance use and co-occurring problems: a latent class analysis. AIDS Behav. 2021;25(8):2501–16. 10.1007/s10461-021-03212-0. 33683531 10.1007/s10461-021-03212-0 PMC7937776

[5] Lancaster KE, et al. Substance use and universal access to HIV testing and treatment in sub-Saharan Africa: implications and research priorities. J Virus Erad. 2018;4(Suppl 2):26–32. 30515311 10.1016/S2055-6640(20)30342-3 PMC6248849

[6] Mohamed I, Ahmad H, Hassaan S, Hassan S. Assessment of anxiety and depression among substance use disorder patients: a case-control study. Middle East Curr Psychiatry. 2020. 10.1186/s43045-020-00029-w.

[7] Pence BW, et al. Association of increased chronicity of depression with HIV appointment attendance, treatment failure, and mortality among HIV-infected adults in the United States. JAMA Psychiatry. 2018;75(4):379–85. 10.1001/jamapsychiatry.2017.4726. 29466531 10.1001/jamapsychiatry.2017.4726 PMC5875308

[8] Mannes ZL, Hearn LE, Zhou Z, Janelle JW, Cook RL, Ennis N. The association between symptoms of generalized anxiety disorder and appointment adherence, overnight hospitalization, and emergency department/urgent care visits among adults living with HIV enrolled in care. J Behav Med. 2019;42(2):330–41. 10.1007/s10865-018-9988-6. 30387009 10.1007/s10865-018-9988-6 PMC6447438

[9] Pelton M, et al. Rates and risk factors for suicidal ideation, suicide attempts and suicide deaths in persons with HIV: a systematic review and meta-analysis. Gen Psychiatr. 2021;34(2): e100247. 10.1136/gpsych-2020-100247. 33912798 10.1136/gpsych-2020-100247 PMC8042999

[10] Carrico AW, et al. Affect regulation, stimulant use, and viral load among HIV-positive persons on anti-retroviral therapy. Psychosom Med. 2007;69(8):785–92. 10.1097/PSY.0b013e318157b142. 17942835 10.1097/PSY.0b013e318157b142

[11] Lynch F, et al. Substance use disorders and risk of suicide in a general US population: a case control study. Addicti Sci Clin Pract. 2020. 10.1186/s13722-020-0181-1. 10.1186/s13722-020-0181-1 PMC7035727 32085800

[12] Abaver DT, Cishe EN. Violence, abuse and discrimination: key factors militating against control of HIV/AIDS among the LGBTI sector. SAHARA J. 2018;15(1):60–70. 10.1080/17290376.2018.1492960. 30025496 10.1080/17290376.2018.1492960 PMC6060376

[13] Nicholson HL, Wheeler PB, Smith NC, Alawode OA. Examining the relationship between discrimination and prescription drug misuse: findings from a national survey of Black Americans. Subst Use Misuse. 2022;57(7):1014–21. 10.1080/10826084.2022.2052096. 35395923 10.1080/10826084.2022.2052096

[14] Matsuzaka S, Knapp M. Anti-racism and substance use treatment: addiction does not discriminate, but do we? J Ethn Subst Abuse. 2020;19(4):567–93. 10.1080/15332640.2018.1548323. 30642230 10.1080/15332640.2018.1548323

[15] Dobkin PL, Civita MD, Paraherakis A, Gill K. The role of functional social support in treatment retention and outcomes among outpatient adult substance abusers. Addiction. 2002;97(3):347–56. 11964111 10.1046/j.1360-0443.2002.00083.x

[16] Reid R, Dale SK. Moderating effects of social support on the relationship between substance use disorders and HIV viral load and medication adherence among Black women living with HIV in the United States. AIDS Care. 2022;34(10):1219–28. 34783618 10.1080/09540121.2021.2001415 PMC9453849

[17] Muthén B, Muthén LK. Integrating person-centered and variable-centered analyses: growth mixture modeling with latent trajectory classes. Alcohol Clin Exp Res. 2000;24(6):882–91. 10888079

[18] Starks TJ, Millar BM, Eggleston JJ, Parsons JT. Syndemic factors associated with HIV risk for gay and bisexual men: comparing latent class and latent factor modeling. AIDS Behav. 2014;18(11):2075–9. 10.1007/s10461-014-0841-9. 25055765 10.1007/s10461-014-0841-9

[19] Henry BF, et al. Typologies of stressful life events and their association with sexual risk behaviors and communication among justice-involved males and their female sex partners. AIDS Educ Prev. 2022;34(5):379–94. 10.1521/aeap.2022.34.5.379. 36181499 10.1521/aeap.2022.34.5.379 PMC9576004

[20] Shokoohi M, et al. Patterns of social determinants of health associated with drug use among women living with HIV in Canada: a latent class analysis. Addiction. 2019;114(7):1214–24. 10.1111/add.14566. 30698902 10.1111/add.14566 PMC6992379

[21] Traynor SM, et al. Differential effects of patient navigation across latent profiles of barriers to care among people living with HIV and comorbid conditions. J Clin Med. 2023. 10.3390/jcm12010114. 10.3390/jcm12010114 PMC9820894 36614917

[22] Metsch LR, et al. Effect of patient navigation with or without financial incentives on viral suppression among hospitalized patients with HIV infection and substance use: a randomized clinical trial. JAMA. 2016;316(2):156–70. 10.1001/jama.2016.8914. 27404184 10.1001/jama.2016.8914 PMC5339876

[23] Metsch LR, et al. Care facilitation advances movement along the hepatitis C care continuum for persons with human immunodeficiency virus, hepatitis C, and substance use: a randomized clinical trial (CTN-0064). Open Forum Infect Dis. 2021;8(8): ofab334. 10.1093/ofid/ofab334. 34377726 10.1093/ofid/ofab334 PMC8339611

[24] Derogatis LR. BSI 18, brief symptom inventory 18: administration, scoring, and procedures manual. Iowa: NCS Pearson Incoportated; 2001.

[25] Trivedi MH, et al. Concise health risk tracking scale: a brief self-report and clinician rating of suicidal risk. J Clin Psychiatry. 2011;72(6):757–64. 10.4088/JCP.11m06837. 21733476 10.4088/JCP.11m06837

[26] Fleishman JA, et al. Coping, conflictual social interactions, social support, and mood among HIV-infected persons. HCSUS Consortium. Am J Commun Psychol. 2000;28(4):421–53. 10.1023/a:1005132430171. 10.1023/a:1005132430171 10965385

[27] Aadil M, Cosme RM, Chernaik J. Mindfulness-based cognitive behavioral therapy as an adjunct treatment of attention deficit hyperactivity disorder in young adults: a literature review. Cureus. 2017;9(5): e1269. 10.7759/cureus.1269. 28775916 10.7759/cureus.1269 PMC5526699

[28] Thompson HS, Valdimarsdottir HB, Winkel G, Jandorf L, Redd W. The group-based medical mistrust scale: psychometric properties and association with breast cancer screening. Prev Med. 2004;38(2):209–18. 10.1016/j.ypmed.2003.09.041. 14715214 10.1016/j.ypmed.2003.09.041

[29] Philbin MM, et al. The North-South divide: substance use risk, care engagement, and viral suppression among hospitalized human immunodeficiency virus-infected patients in 11 US Cities. Clin Infect Dis. 2019;68(1):146–9. 10.1093/cid/ciy506. 29920584 10.1093/cid/ciy506 PMC6293003

[30] Webb P, Coates J, Frongillo EA, Rogers BL, Swindale A, Bilinsky P. Measuring household food insecurity: why it’s so important and yet so difficult to do. J Nutr. 2006;136(5):1404S-1408S. 10.1093/jn/136.5.1404S. 16614437 10.1093/jn/136.5.1404S

[31] McLellan AT, et al. The fifth edition of the addiction severity index. J Subst Abuse Treat. 1992;9(3):199–213. 10.1016/0740-5472(92)90062-s. 1334156 10.1016/0740-5472(92)90062-s

[32] Saunders JB, Aasland OG, Babor TF, de la Fuente JR, Grant M. Development of the alcohol use disorders identification test (AUDIT): WHO collaborative project on early detection of persons with harmful alcohol consumption–II. Addiction. 1993;88(6):791–804. 10.1111/j.1360-0443.1993.tb02093.x. 8329970 10.1111/j.1360-0443.1993.tb02093.x

[33] Skinner HA. The drug abuse screening test. Addict Behav. 1982;7(4):363–71. 10.1016/0306-4603(82)90005-3. 7183189 10.1016/0306-4603(82)90005-3

[34] Yuan K-H, Bentler P. 5. Three likelihood-based methods for mean and covariance structure analysis with nonnormal missing data. Sociol Methodol. 2000;30(1):165–200. 10.1111/0081-1750.00078.

[35] Nylund K, Asparouhov T, Muthén B. Deciding on the number of classes in latent class analysis and growth mixture modeling: a Monte Carlo simulation study. Struct Equ Modeling. 2007;14(4):535–69. 10.1080/10705510701575396.

[36] Muthén LK, Muthén B. Mplus: the comprehensive modeling program for applied researchers: user’s guide. Los Angeles: Muthén & Muthén©; 1998.

[37] SAS. SAS/IML® 14.1 user’s guide. Cary: SAS Institute Inc; 2015.

[38] Yehia BR, et al. Health outcomes of HIV-infected people with mental illness. AIDS Behav. 2015;19(8):1491–500. 10.1007/s10461-015-1080-4. 25931243 10.1007/s10461-015-1080-4 PMC4527875

[39] CDC. Monitoring selected national HIV prevention and care objectives by using HIV surveillance data—United States and 6 dependent areas, 2021. In: HIV Surveillance Supplemental Report. vol. 28. 2023. https://www.cdc.gov/hiv/group/racialethnic/other-races/viral-suppression.html. Accessed on 1 Nov 2023

[40] Matacotta JJ, Tran D, Yoon S. The prevalence of major depressive disorder in people with HIV: results from the all of us research program. HIV Med. 2024. 10.1111/hiv.13653. 38715437 10.1111/hiv.13653 PMC12711201

[41] van Luenen S, Garnefski N, Spinhoven P, Spaan P, Dusseldorp E, Kraaij V. The benefits of psychosocial interventions for mental health in people living with HIV: a systematic review and meta-analysis. AIDS Behav. 2018;22(1):9–42. 10.1007/s10461-017-1757-y. 28361453 10.1007/s10461-017-1757-y PMC5758656

[42] Byrd KK, et al. Improvements in retention in care and HIV viral suppression among persons with HIV and comorbid mental health conditions: patient-centered HIV care model. AIDS Behav. 2020;24(12):3522–32. 10.1007/s10461-020-02913-2. 32415615 10.1007/s10461-020-02913-2 PMC7666642

[43] Griffin A, et al. Addressing disparities in the health of persons with HIV attributable to unstable housing in the United States: the role of the Ryan White HIV/AIDS Program. PLoS Med. 2020;17(3): e1003057. 10.1371/journal.pmed.1003057. 32119661 10.1371/journal.pmed.1003057 PMC7051042

